# The role of p53, PCNA and Ki–67 as outcome predictors in the treatment of laryngeal cancer

**Published:** 2009-04-25

**Authors:** D Sarafoleanu, V Postelnicu, C Iosif, C Manea, C Sarafoleanu

**Affiliations:** * ‘Sfanta Maria’Clinical Hospital, ENT– HNS Department, Bucharest Romania; ** ‘Victor Babes’Institute, Bucharest, Romania

## Abstract

The aim of our study was to determine the importance of p53, PCNA and Ki–67, evaluated by immunohistochemistry, in the treatment and prediction of the laryngeal carcinoma. Out of a total of 319 patients with laryngeal carcinoma that underwent surgery in our department between 1999 and 2007, we performed a retrospective study on 71 cases who benefited by immunohistochemical guidance before thebeginning of the treatment. All these patients have been followed–up two to five years after surgery. The values of p53, PCNA and Ki–67 are strongly correlated with the histological grading, by means of descriptive statistics (confidence level 95%); the mean values of these three markers corresponding to each HP grade. A highly statistical significant positive correlation (r = 0.84, p<0.001) between the values of p53 and PCNA was observed. The values of p53, PCNA and Ki–67 in the patients from this study are strongly correlated with the absence of the loco–regional lymph node metastases, by means of descriptive statistics(confidence level 95%). Ki–67 only is correlated significantly to the presence of lymphatic metastases in the regional lymph nodes (stage N1, N2 or N3 TNM). P53 and PCNA are not correlated significantly with the presence of the metastases in the regional lymph nodes.

## Introduction

We will comment upon several aspects of a clinical study regarding the p53, PCNA and Ki–67 expression in laryngeal cancers. The aim of our study was to determine the importance of these three biological markers, evaluated by immunohistochemistry, in the treatment and prediction of the laryngeal carcinoma.

The squamous cell carcinoma represents about 90% of malignant tumors of the larynx, being correlated with smoking and alcoholism. The laryngeal malignancies are more common in men aged 55 to 70, but women and children are not excluded; laryngeal cancer is known to be responsible for 0.9% of the total deaths from cancer and is one of the most curable malignancy of this region, because most of them are early diagnosed. Laryngeal cancer cases may manifest local rebound or metastasis in the regional lymph nodes, determining a significant morbidity and mortality. Although the clinical staging is an important outcome predictor, there are many genetic mutations of the onco–genes and of the tumor–suppressor genes which interfere with tumoral growth and these genetic influences are insufficiently studied[1,2, 3].

These tumors can be classified anatomically, depending on their position in the larynx: supraglottic, glottic and subglottic cancers. The glottic localization has the best prognosis because of its poor lymphatic drainage, slow tumoral progression and late metastasis. A particular situation is found in the hypopharyngeal cancer which has lesions located in the piriform sinus and in the postcricoidian region.

The histological classification is made by the following criteria: the cellular differentiation, pleiomorphism and architectural abnormalities, the number of mitosis and the tumor–host interaction.

For a better understanding of the growth and progression of the pre–neoplastic and neoplastic lesions of the superior aero–digestive tract, we studied factors with previously demonstrated prognostic value in other cancers, like p53, PCNA and Ki–67. The p53 protein is present in all normal cells, but the half life of the ‘wild’ (normal) protein is so short (6–30min) that does not reach high enough levels in order to be detected by the standard immunohistochemical techniques. On the other side, the mutant p53 has a much higher half life, so it accumulates and is detected in the cellular nucleus. The alteration of the p53 creates a mutant p53, which is also expressed, but does not have the regulatory function as the ‘wild’ p53 does. The p53 positive cells are those that suffered mutations of the p53 gene. Proliferation cell nuclear antigen, PCNA is a nuclear antigen present in the G1 and S phases of the cellular cycle. Low values of PCNA are found in the basal stratus of the pavimentous epithelia. Ki–67 also a nuclear antigen present in the active phases of the cellular cycle (G1, S, G2, M and absent in G0), thus reflecting cellular division; the tumors which are in cellular division but spend more time than normal will over–express the Ki–67 antigen. The protein of the p53 oncogene (situated on the Cz17p chromosome) is an onco–suppressive protein which monitors the cellular cycle. A positive correlation between the high proliferation rate and abnormality of p53 is described in literature [4–24]. 

## Material and methods

Out of a total of 319 patients with laryngeal carcinoma operated in our clinic between 1999 and 2007, we performed a retrospective study on 71 cases who benefited by immunohistochemical guidance before the beginning of the treatment. All these patients have been followed–up two to five years after surgery.

The tumoral lesions were isolated using laryngeal endoscopy by debulking biopsy using classical or carbon dioxide laser resection. The biopsy samples were formalin fixed, paraffin embedded and examined by histopathology and immunohistochemistry (IHC) at the ‘Victor Babes’ Institute, Bucharest, Romania. To ensure the reliability of the experimental study, internal quality control of IHC techniques was performed as a part of an implemented and certified quality assurance system (ISO 15189/2007).

**Figure 1:**
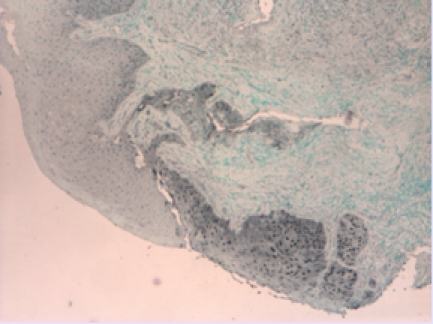
p53+ ~ 30–40% in carcinoma in situ; P53+ ~  5% in the areas with simple dysplasia; IHC, 4x.

The procedure employed consisted in deparaffinization in xylene and alcohol series, rehydration, washing in phosphate saline buffer (PBS), incubation with normal serum, for 20 minutes, incubation with primary antibody overnight, standard labeled streptavidine–antibody biotin (LSAB) kit (DAKO), washing in carbonate buffer and development in 3–3'–DAB hydrochloride / H2O2; immunostain amplification with heavy metals (cobalt) was performed for nuclear antigens. The routine stain used was Haematoxylin and Eosin (H&E) for the assessment of the histopathological aspects and the mitotic index. The IHC was performed on 3 micro m thick sections from 10% formalin fixed paraffin embedded tissues, according to the indirect tristadial Avidin–Biotin–Complex Peroxidase method of HSU et al [25], modified by Bussolati and Gugliotta[26]. The selected cases were tested by IHC using the following antibodies: PCNA – clone PC10, dilution 1:200, kit source DAKO and p53–clone DO7, dilution 1:50, kit source Neomarkers. All specimens were counterstained with Meyer's haematoxylin, examined and photographed on a Nikon Eclipse 600 microscope (Figure 1–Figure 6 display examples of the obtained result).

**Figure 2:**
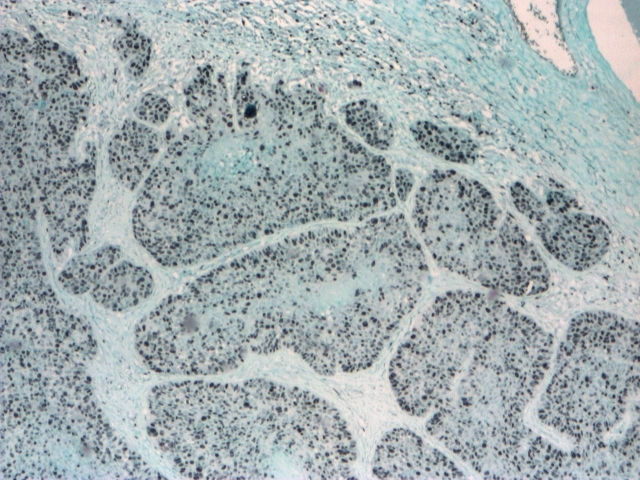
p53+ ~  60% in invasive undifferentiated squamous cell carcinoma; p53+ ~ 1% in the residual papillomatous areas; IHC, 4x.

**Figure 3:**
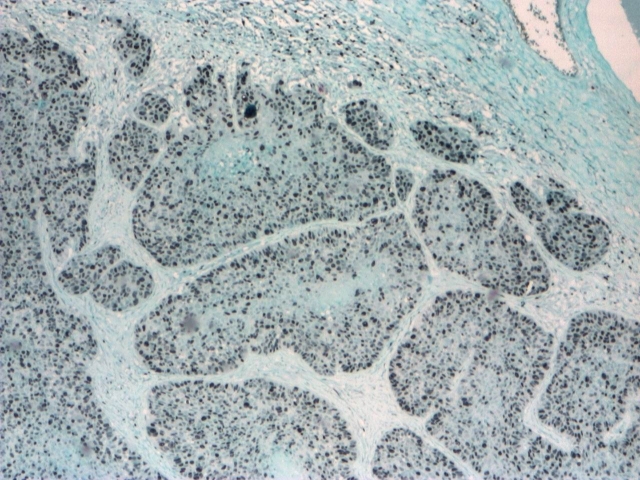
PCNA+ ~  40–50% in moderate differentiated squamous cell carcinoma; PCNA+ ~ 10% at the invasion front; IHC, 4x.

**Figure 4:**
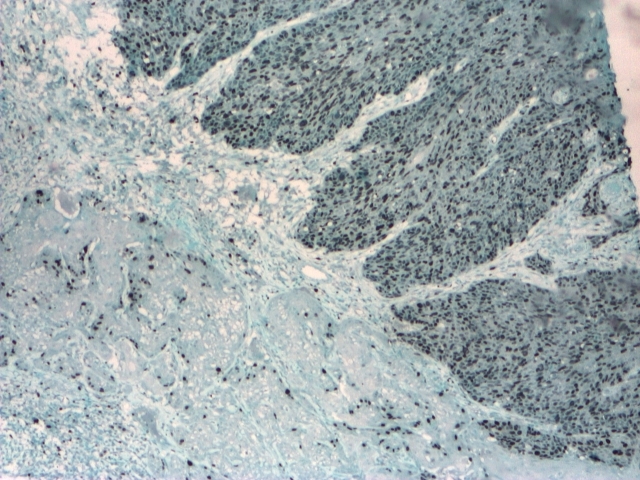
PCNA+ ~  60–80% in the areas with poor differentiated basaloid carcinoma; PCNA+ 3–5% in the areas with moderate differentiated squamous carcinoma; IHC, 4x.

**Figure 5:**
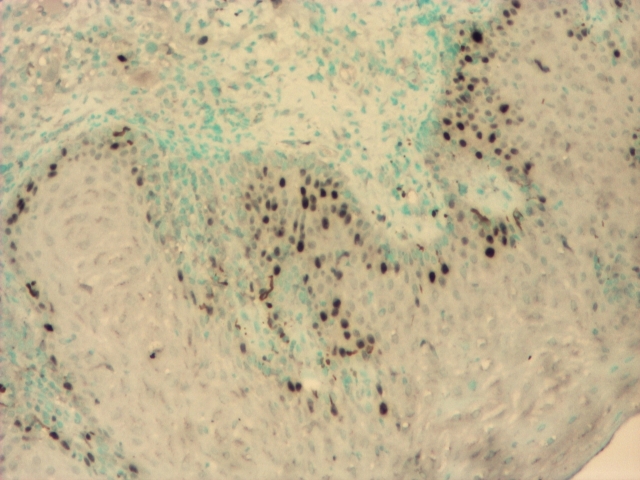
Ki–67+ ~  20% in the inferior 1/3 of the squamous epithelium with simple dysplasia; IHC, 10x;

**Figure 6:**
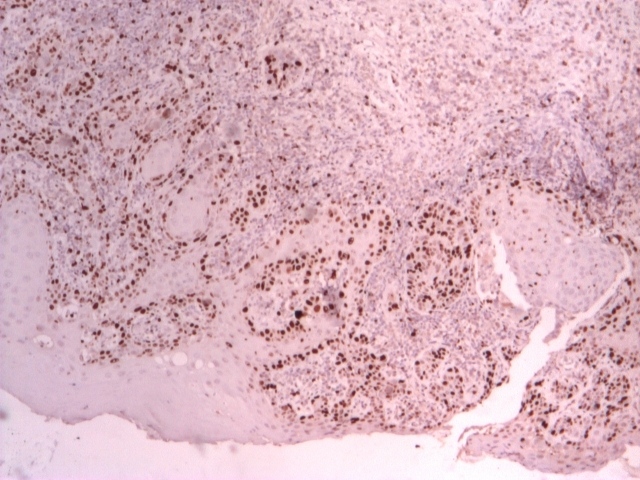
Ki–67+ in the basal stratus and ~  40% in islands of micro–invasive squamous cell carcinoma and carcinoma in situ; IHC, 4x.

The patients included in this study were aged between 35 and 71 years and the majority (90.1%) were men. The highest incidence of the laryngeal carcinoma was identified in the 6th decade of life–43.7% of cases, followed by the 7th decade–29.6% and the 5th decade–19.7%. The lowest incidence of the laryngeal carcinoma in this study was described over 71 years–4.2% and under 40 years old–2.8% of cases.

Regarding the clinical staging, the majority of the patients were diagnosed in advanced TNM stages–30.99% in stages IV TNM and 26.76% in stages 3 TNM. 36.6% of the patients had metastases in the regional lymph nodes (stages N1, N2 and N3 TNM) at the time of diagnosis. The glottic invasion was found in 91.5% of the patients, supraglottic invasion in 31% and subglottic extension in 11.3% of the patients. The histology was well to moderate differentiated in 67.6% of the tumors and poor differentiated or undifferentiated in 32.4% of the tumors.

The surgical procedures applied after histological investigations were: 52.1% of the patients underwent total laryngectomy, 40.9% carbon dioxide laser resection of the tumoral lesions and 7% partial laryngectomy.

After the IHC analysis of the initial biopsies and of the resection specimens, (margins of resection, regional lymph node metastases, TNM staging), the operated patients underwent IHC guided radiotherapy. Radiotherapy was applied to all the patients with metastases in the regional lymph nodes and to 42.2% of the patients without regional lymph node metastases. Statistically, IHC guided radiotherapy was applied to all patients diagnosed in stage Ⅳ TNM, as well as to 57.9% of those diagnosed in stage Ⅲ TNM, 42.9% of those diagnosed in stage Ⅱ TNM and 33.3% of the patients diagnosed in stage Ⅰ TNM.

## Results

The majority of the patients with squamous laryngeal carcinoma from this study survived after IHC guided surgery and radiotherapy – 66.2%. The tumoral rebounds and regional metastases after surgery were found in approx. 19% in the first six months after surgery, in 32% in the first two years and 49% in the first five years.

**Chart 1:**
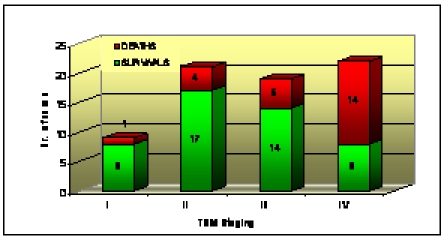
Survival versus TNM staging

Regarding the TNM staging, the highest survival rates were identified in the stage Ⅰ TNM –88.9%, in the stage Ⅱ TNM – 80.9% and 73.7% of the patients survived. The highest mortality rate was identified in the stage Ⅳ TNM – 63.6% of the patients included in this study (as shown in Chart 1).

The highest survival rates were obtained for well differentiated laryngeal carcinomas – 90% of the patients and for the moderate differentiated laryngeal carcinomas–71.05%; the lowest survival rates had the poor differentiated or undifferentiated laryngeal carcinomas–47.8% of the patients survived.

**Chart 2:**
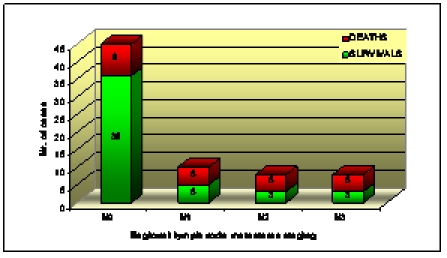
Survival versus regional lymph node

The majority of the patients without regional lymph node metastases–76.6% of those diagnosed in stage N0 TNM–survived. The lowest survival rates were obtained for the patients diagnosed in stage N1 TNM–50%, in stage N2 and N3 TNM–only 37.5% for each stage. Thus, the highest mortality rates are found in the patients with regional lymph nodes metastases–57.7% deceased in the first two to fives years after IHC guided surgery and radiotherapy (as shown in Chart 2).

The correlation between the values of p53, PCNA, Ki–67 the histological grading. The values of p53, PCNA and Ki–67 are strongly correlated with the histological grading, by means of descriptive statistics (confidence level 95%); the mean values of these three markers corresponding to each HP grade are shown in the Table 1.

**Table 1:** IHC correlation with the HP grade.

HP Grade	Mean values of IHC predictors in SCLC compared with the HP Grade
	p53 (%)	PCNA (%)	Ki–67 (%)
Well differentiated	13.5	37	13
Moderate differentiated	19.2	45.1	14.9
Poor differentiated	42.3	67.8	23.7

**Chart 3:**
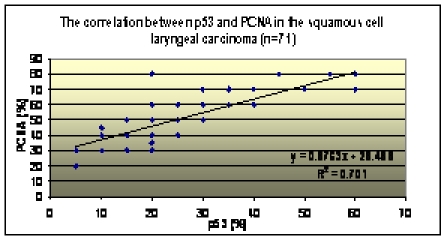
p53–PCNA correlation

**Chart 4:**
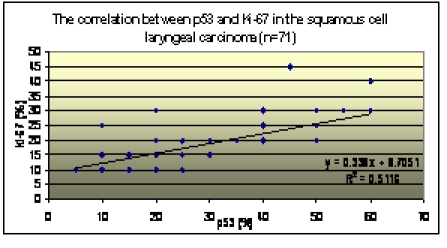
p53–Ki–67 correlation

The correlation between the values of p53, PCNA and Ki–67. A highly statistical significant positive correlation (r = 0.84, p<0.001) between the values of p53 and PCNA was observed. The values of p53 and Ki–67 also have a highly statistical significant positive correlation (r = 0.71, p<0.001), indicating that these three factors correlated have a superior prognostic value when considered together [Chart 3,Chart 4].

The correlation between the values of p53, PCNA, Ki–67 and the local metastases in the regional lymph nodes. The values of p53, PCNA and Ki–67 in the patients from this study are strongly correlated with the absence of the loco-regional lymph node metastases,by means of descriptive statistics (confidence level 95%). Ki–67 only is correlated significantly to the presence of lymphatic metastases in the regional lymph nodes (stage N1, N2 or N3 TNM). P53 and PCNA are not correlated significantly with the presenceof the metastases in the regional lymph nodes (Table 2).

**Table 2:** IHC correlation with the regional lymph node metastases.

Lymph metastases(TNM stage)	Mean values of IHC predictors in SCLC regarding regional lymph node metastases
	p53 (%)	PCNA (%)	Ki–67 (%)
N0	24	49.1	17
N1, N2 or N3	29.2	55	18.3

The correlation between the values of p53, PCNA, Ki–67 and survival. The values of p53, PCNA and Ki–67 are strongly correlatedwith the survival of patients, by means of descriptive statistics (confidence level 95%), with the mean values as shown in the Table 3.

**Table 3:** IHC correlation with the survival status.

Survival status	Mean values of IHC predictors in SCLC regarding the therapeutic outcome
	p53 (%)	PCNA (%)	Ki–67 (%)
Survivals	20.1	45.9	15.7
Deaths	37.3	61.8	20.8

## Discussions

Despite the continuous upgrades in radiotherapy and surgical techniques in the past two decades, the survival rate in superior aero–digestive tract cancers had been little influenced. New predictive factors can nowadays be determined before any treatment or in early stages of the treatment to allow the distribution of patient in subgroup with a particular known evolution, in order to undergo differentiated diagnostic and therapeutic protocols with better results regarding the overall survival.

The evolution speed of the carcinoma and the risk of metastasis are proportional with local lymph node initial invasion. Therewith, an unsolved query for the moment is the fact that at the same level of lymph node invasion, some of the patients develop rapidly metastases whereas others never do.

Laryngeal cancers have diverse types of structure and evolution. This is why, in order to be able to establish the prognostic factors, we must understand the biologic evolution and the natural history of every type of cancer. It is also very important to determine the factors closely related to the patient's organism, to the tumor and to the therapy means we intend to use.

The predictive factors are classified by current oncology studies as follows:

- factors related to the tumor, to the peri–tumoral extension and to the TNM stage;
- histopathological factors: cellular differentiation, mitotic activity, capsular invasion and breakage, inflammatory peri–tumoral infiltrate and cervical lymph nodes invasion;
- biological factors determined by immunohistochemistry: the onco–suppressive gene p53 modifications, PCNA and Ki–67 nuclear antigens, bcl–2 pro–oncogene, EGFR, VEGF and VEGF–C growth factors.

Our study was meant to explore the correlation between the over–expression of p53, PCNA, Ki–67 and the tumoral growth, early lymphatic metastasis, response to surgery / radio–therapy and overall survival.

Based on the statistical sample analyzed, we have shown there is a strong correlation between the tumoral cells with a high rate of DNA synthesis and the expression of PCNA and Ki–67. There is also a strong relationship between the high rate of cellular proliferation and p53 abnormalities.

A well or moderate differentiated tumor associated with PCNA positivism below 40% is a good prognosis of the disease. PCNA higher than 40% and poorly differentiated or undifferentiated tumors are generally correlated with metastasis and local recurrence.

## Conclusions

The biological factors of tumoral aggression correlate well with the overall survival of the patients. Furthermore, due to the fact that normally all three factors analyzed occur together, the correlation of all factors can give a much stronger indication than one factor alone.

High values of positive p53 (20–60%), PCNA (60–80%) and Ki–67 (higher than 40%) are correlated with a high biological aggressiveness of the tumor and with the histological grading, respectively poor differentiated or undifferentiated carcinomas. Moderate values of positive p53 (10–20%), PCNA (40–60%) and Ki–67 (20–40%) are correlated with a moderate biological aggressiveness of the tumor and with the histological grading, respectively moderate differentiated carcinomas. Low values of positive p53 (5–15%), PCNA (20–40%) and Ki–67 (20–40%) are correlated with a moderate biological aggressiveness of the tumor and with the histological grading, respectively good differentiated carcinomas.

The most important clinical predictive factors are the TNM staging, the tumor localization and the age of the patient. The majority of patients studied were diagnosed with advanced stages of laryngeal carcinoma –  stages Ⅲ and Ⅳ TNM – more than 56% of cases. Late diagnosis on this statistical sample has afforded extraction of representative statistical data on the evolution of the disease, which would not otherwise be available. The functional and ‘quo ad vitam’ prognosis depends on the correct interpretation of the predictive factors, the correct staggering of the therapy –  chemotherapy, radiotherapy and surgery – by means of the tumoral grading and staging.

A correct applied radiotherapy, guided by the clinical and immunohistochemical predictive factors may lead to better healings, even for carcinomas diagnosed in the stage Ⅲ TNM without surgical intervention. The chemotherapy is useful in local rebounds or lymphatic metastases after surgery.

We have demonstrated that the treatment guidance after the immunohistochemical predictive factors raises the overall survival by 30–40%. The evaluation of these predictive factors should be made at the moment of diagnosis or in the early stages of the treatment. It is important to note that the probability of re–occurrence can also be evaluated based on these factors, and as such they are an indispensable guide in determining the appropriate treatment strategy.
